# Awareness and support for anti-tobacco policies among health professional students in Pakistan: findings from the Global Health Professional Students Survey, 2011

**DOI:** 10.1186/s13011-015-0001-x

**Published:** 2015-03-08

**Authors:** Syeda Kanwal Aslam, Beenish Mehboob, Sidra Zaheer, Kashif Shafique

**Affiliations:** School of Public Health, Dow University of Health Sciences, OJHA Campus, SUPARCO road, Gulzar e Hijri, Karachi, Pakistan; Institute of Health and Wellbeing, Public Health, University of Glasgow, 1-Lilybank Gardens, G12 8RZ Glasgow, UK

**Keywords:** Smoking, Health professionals, Tobacco, Anti-tobacco policy

## Abstract

**Background:**

Health professional (HP) students may have an important role in controlling future tobacco use of their patients, and public at large. It is important to understand their existing level of awareness and support for national anti-tobacco policies. We thus aim to explore Pakistani HP students’ existing attitudes towards national anti-tobacco policy and examine factors associated with lack of awareness, and support amongst them.

**Methods:**

Secondary data analysis of the Global Health Professional Students Survey, Pakistan, 2011 was performed. Study population included 4,235 health professional students enrolled in third year of graduate level HP programs. The policy support metrics were developed using six questions from the survey. Univariate and multivariate analyses were conducted to analyze association between HP students’ awareness, and support for anti-tobacco policy (outcome variables), and various socio-demographic, attitudinal, and knowledge related factors. Descriptive statistics are reported as proportions, and results of logistic regression analysis were reported as odds ratios with 95% confidence interval.

**Results:**

Overall, among HP students, 10.8% (n = 391) were current smokers, and 26.7% (n = 965) of them were cigarette experimenters. Almost half, (46.1%, n = 1666) of the HP students did not have an awareness of the official policy banning tobacco use in their school buildings and clinics; and only one in ten (9.4%, n = 338) of them did not support anti-tobacco policies. Students were less likely to be aware if they had second hand exposure at home/work (OR = 0.73, 95% CI (0.57-0.92), p-value <0.01). Furthermore, students who were current smokers (OR = 0.21, 95% CI (0.08-0.56), or cigarette experimenters (OR = 0.42, 95% CI (0.26-0.70), p-value <0.01), were least likely to support anti-tobacco policies.

**Conclusion:**

We found that HP students lack awareness of anti-tobacco policies; and were less likely to support such efforts if they were current smokers. These findings may help in understanding existing perceptions of the future care givers in Pakistan. Future anti-tobacco efforts and HP training programs may target the smoking HPs to enhance their full support in this regard.

**Electronic supplementary material:**

The online version of this article (doi:10.1186/s13011-015-0001-x) contains supplementary material, which is available to authorized users.

## Background

Tobacco smoking is a leading cause of premature mortality all over the world causing more than five million annual deaths [1]. Recent trends report a higher threat to the health of populations in developing countries, including Pakistan; where almost 17% of all adults are current smokers [2]. In order to prevent tobacco use among masses, many countries have implemented strong anti-tobacco policies. Such policies include increasing tax on tobacco products, restricting sale of tobacco to the adolescents, and strict enforcement of tobacco bans at public places [3]. Such efforts have resulted in considerable decline in tobacco use prevalence [4]. However several reports reveal that developing countries like Pakistan, despite having a policy ban on smoking, still have very high tobacco prevalence, mainly due to weak anti-tobacco legislative implementation [5].

Research literature suggests that increasing awareness regarding tobacco policies may result in decreasing tobacco use acceptability among masses [6,7]. These effects may be influenced by various cultural factors, and thus it may be helpful to identify particular behavioral and attitudinal aspects that determine the extent to which such policies are enforced in a society [8]. Additionally, smoking status may influence an individual’s attitude towards the anti-tobacco policies [9]. It is suggested that anti-tobacco policies may be received with resentment among smokers, as they might perceive that such bans limit their individual freedom [10]. Furthermore, according to the “Reactance theory” of behavior, smoking might gain more attraction among adolescents, who wish to reassert their personal autonomy by rebelling against such bans [11]. However, little is known about these attitudinal aspects in our context.

Health professionals (HP) play an important role in tobacco control, and have been regarded as the main task force in controlling tobacco use among masses. According to the WHO Framework Convention on Tobacco Control’s (WHO FCTC) Article 12, all member countries are encouraged to implement effective tobacco use control programs for HPs and HP students; and MPOWER report states that the primary responsibility of managing tobacco dependence lies with any country’s healthcare system [12]. Unfortunately, HP students in many developing countries are not well prepared to take up the desired role. They do not receive adequate training in this regard, do not perceive that HPs have a role in tobacco control, and are themselves the victims of tobacco use [13,14]. It may be due to the unfamiliarity with anti-smoking policy that they neither advocate for such policy nor perceive themselves as a role model for the society. Currently, little is known about the attitudes of Pakistani HP students towards anti-tobacco policies. We aim to explore Pakistani HP students’ existing attitudes towards national anti-tobacco policy and examine factors associated with lack of awareness, and support amongst them.

## Methods

### Data

We used nationally representative data on health professional students from Pakistan. It was collected through the Global Health Professional Students Survey (GHPSS) Pakistan, 2011; conducted by the World Health Organization (WHO), and Centers for Disease Control and Prevention (CDC). GHPSS followed standardized methods for data collection and analysis which are detailed in country specific report of GHPSS, 2011 [15]. Local procedures for obtaining ethical approval were followed. It used multistage sample design: institutes were selected proportional to enrollment size, classes were selected randomly within institutes; and all students of the selected classes were eligible to participate.

### Participants

Participants were third year students enrolled in graduate level degree programs of medical, dental and pharmacy professions.

### Data collection tool

GHPSS uses anonymous, self-administered questionnaire, which includes information on demographics, tobacco use, tobacco related knowledge, attitudes and perceptions. For this study, we selected questions related to tobacco use, anti-tobacco policy support and awareness.

### Variables

Awareness of anti-tobacco policies, and support for anti-tobacco policies were used as dependent variables. Support for anti-tobacco policies was developed by using information related to six policy metrics. Independent variables included: age, sex, smoking status, health professional degree program, second hand smoke exposure, perception of HP as role model for patients and public, perceived role of HP in tobacco control, received training about dangers of tobacco use. Detail about all variables included in the study, and operationalization of variables is available in the online supplement (Additional file 1: Table S1).

### Analysis

SAS version 9.1.3. is used for conducting analysis. To account for the multistage cluster sampling design, complex survey data analysis was used; and primary sampling units, final weights and strata were used to adjust for weighted analysis. Firstly, descriptive of the sample were reported as frequency and percentage. Secondly, the Chi-square test of association between current smoking status and all study variables were conducted, and p-values less than 0.05 were taken as significant. Lastly univariate and multivariate analyses were conducted between all study variables and the dependent variables: awareness of policies and support for policies; and the associations were reported as odds ratios and 95% confidence intervals. During multivariate regression analysis, we adjusted for various factors including: age, sex, health profession, smoking status, second hand exposure to smoking, perceptions as role model for patient and public, perceived role of HP in tobacco control, training about dangers of tobacco use.

## Results

The study sample included 4,235 undergraduate HP students, from 8 dental, 21medical and 15 pharmacy institutions of Pakistan. We constructed the dataset using information from 3612 participants, dental (n = 340), medical (n = 2351) and pharmacy students (n = 921) after excluding 641 participants due to missing data. The overall student’s response rates were 70.3%, 69.2% and 70.4% for dental, medical and pharmacy programs respectively.

Overall, 10.8% (n = 391) of HP students were current smokers, and 26.7% (n = 965) were cigarette experimenters. Majority of current smokers (88%) and cigarette experimenters (67.4%) were males. We found that 46.1% (n = 1666) of the HP students did not have an awareness of an official policy banning tobacco use in their school buildings and clinics. The smoking students were least aware of such institutional policies (smokers = 34.8%, smokers who attempted to quit smoking 40.7%, experimenters = 40.9%, ex-smokers = 43.7% and never smokers = 47.3%) (Additional file 2: Table S2).

Furthermore, we looked at six support policy metrics among the HP students according to their smoking status.

### Ban on tobacco sale to individuals younger than 18 years of age

Overall, 19.9% of smoking students, 15.6% of cigarette experimenters, and 15% of smokers who had attempted to quit smoking reported no support for this ban. Never smoking students reported the highest support for this ban (Additional file 2: Table S2).

### Ban on tobacco advertisements

Furthermore, 26.1% of the smoking HP students did not support ban on tobacco advertisements, followed by experimenter (18.4%). Never smokers reported highest support for this ban. The results of other policy metrics were also comparable with these findings, for complete results see Additional file 2: Table S2.

Chi-square test results indicate that students’ awareness of anti-tobacco policies (χ^2^ = 31.01, *df* = 1, p-value < 0.01), support for anti-tobacco policies (χ^2^ = 244.20, *df* = 1, p-value < 0.01), age (χ^2^ = 160.10, *df* = 2, p-value < 0.01), sex (χ^2^ = 122.0, *df* = 1, p-value < 0.01), health profession (χ^2^ = 38.70, *df* = 2, p-value < 0.01), second hand smoke exposure (χ^2^ = 331.40, *df* = 1, p-value < 0.01), and perception of HP as a role model for patients and public (χ^2^ = 43.29, *df* = 1, p-value < 0.01) were significantly associated with their current smoking status (Table 1).

**Table 1:** **Descriptive characteristics of health professional students by their current smoking status (n = 3612)**

**Characteristics**		**Never-smoker**	**Ex-smoker**	**Experimenter**	**Current smoker**	**Smoker who attempted to quit**	
		**n = 2820**	**n = 396**	**n = 965**	**n = 391**	**n = 349**	
		**n (%)**	**n (%)**	**n (%)**	**n (%)**	**n (%)**	p-value*
**Anti-tobacco policy awareness**							
	No	1334 (47.3)	173 (43.7)	395 (40.9)	136 (34.8)	142 (40.7)	< 0.01
	Yes	1486 (52.7)	223 (56.3)	570 (59.1)	255 (65.2)	207 (59.3)	
**Anti-tobacco policy support**							
	No	180 (6.4)	45 (11.4)	184 (19.1)	111 (28.4)	63 (18.1)	< 0.01
	Yes	2640 (93.6)	351 (88.6)	781 (80.9)	280 (71.6)	286 (81.9)	
**Age in years (categorical)**							
	15-18	152 (5.4)	9 (2.3)	39 (4.0)	23 (5.9)	10 (2.9)	< 0.01
	19-24	2627 (93.2)	381 (96.2)	880 (91.2)	330 (84.4)	301 (86.2)	
	≥25	41 (1.5)	6 (1.5)	46 (4.8)	38 (9.7)	38 (10.9)	
**Sex**							
	Female	2109 (74.8)	137 (34.6)	315 (32.6)	47 (12.0)	52 (14.9)	<0.01
	Male	711 (25.2)	259 (65.4)	650 (67.4)	344 (88.0)	297 (85.1)	
**Health profession**							
	Dental	277 (9.8)	30 (7.6)	81 (8.4)	32 (8.2)	24 (6.9)	<0.01
	Medical	1840 (65.2)	284 (71.7)	645 (66.8)	245 (62.6)	194 (55.6)	
	Pharmacy	703 (25.0)	82 (20.7)	239 (24.8)	114 (29.2)	131 (37.5)	
**SHS exposure**							
	No	1116 (39.6)	111 (28.0)	205 (21.2)	24 (6.1)	34 (9.7)	<0.01
	Yes	1704 (60.4)	285 (72.0)	760 (78.8)	367 (93.9)	315 (90.3)	
**Perception: HP is a role model for patients and public**							
	No	564 (20.0)	86 (21.7)	255 (26.4)	117 (29.9)	107 (30.7)	<0.01
	Yes	2256 (80.0)	310 (78.3)	710 (73.6)	274 (70.1)	242 (69.3)	
**Perception: HP has a role in tobacco control**							
	No	334 (11.8)	41 (10.4)	134 (13.9)	50 (12.8)	49 (14.0)	0.26
	Yes	2486 (88.2)	355 (89.6)	831 (86.1)	341 (87.2)	300 (86.0)	
**Received training about tobacco health effects**							
	No	732 (26.0)	106 (26.8)	270 (28.0)	105 (26.9)	75 (21.5)	0.21
	Yes	2088 (74.0)	290 (73.2)	695 (72.0)	286 (73.1)	274 (78.5)	

* The p-value has been calculated using Chi-square test. p-value of <0.05 is taken as significant.

### Awareness of Anti-tobacco policies

Univariate analysis indicates that students who: were smokers (OR = 1.77, 95% CI (1.05-2.98), p-value 0.03), and received training about dangers of tobacco use (OR = 1.56, 95% CI (1.02-2.38), p-value 0.03), were more likely to be aware of anti-tobacco policies. Students were less likely to be aware of these policies if they had second hand exposure of smoking (OR = 0.76, 95% CI (0.61-0.93), p-value 0.01). Furthermore, after adjusting for all study variables, multivariate analysis indicates that students who had second hand exposure were less likely to be aware of anti-tobacco policies (OR = 0.73, 95% CI (0.57-0.92), p-value <0.01); meanwhile the students who received training about dangers of tobacco use were more likely to have awareness (OR = 1.62, 95% CI (1.05-2.50), p-value 0.02) (Table 2).

**Table 2:** **Factors associated with awareness, and support for anti-tobacco policies among HP students (n = 3612)**

**Characteristics**	**Anti-tobacco policy awareness**				**Anti-tobacco policy support**			
	**OR (95% CI)**	**p-value**	**AOR (95% CI)**	**p-value**	**OR (95% CI)**	**p-value**	**AOR (95% CI)**	**p-value**
**Smoking status**								
Never smoker	1		1		1		1	
Ex-smoker	1.11 (0.76-1.60)	0.58	0.87 (0.44-1.70)	0.68	0.46 (0.34-0.63)	<0.01	1.08 (0.43-2.70)	0.85
Experimenter	1.03 (0.85-1.23)	0.77	1.03 (0.82-1.30)	0.77	0.36 (0.24-0.52)	<0.01	0.42 (0.26-0.70)	<0.01
Current smoker	1.77 (1.05-2.98)	0.03	1.72 (0.86-3.44)	0.12	0.11 (0.06-0.17)	<0.01	0.21 (0.08-0.56)	0.01
Smoker who attempted to quit	1.29 (0.82-2.01)	0.25	1.04 (0.59-1.83)	0.87	0.27 (0.18-0.39)	<0.01	0.74 (0.34-1.62)	0.45
**Age in years (categorical)**								
15-18	1		1		1		1	
19-24	0.66 (0.43-1.02)	0.06	0.71 (0.49-1.04)	0.07	1.90 (0.72-5.01)	0.19	1.84 (1.12-3.04)	0.01
≥25	0.87 (0.37-2.04)	0.76	0.83 (0.38-1.80)	0.64	0.86 (0.29-2.57)	0.79	1.87 (0.86-4.07)	0.11
**Sex**								
Female	1		1		1		1	
Male	1.23 (0.86-1.73)	0.24	1.16 (0.82-1.63)	0.39	0.46 (0.33-0.63)	<0.01	0.95 (0.69-1.31)	0.77
**Health profession**								
Dental	1		1		1		1	
Medical	1.21 (0.64-2.26)	0.54	1.27 (0.65-2.48)	0.48	1.41 (0.91-2.17)	0.12	1.61 (0.88-2.95)	0.12
Pharmacy	1.09 (0.57-2.07)	0.78	1.15 (0.58-2.29)	0.68	0.95 (0.49-1.81)	0.88	1.19 (0.54-2.59)	0.66
**SHS exposure**								
No	1		1		1		1	
Yes	0.76 (0.61-0.93)	0.01	0.73 (0.57-0.92)	<0.01	0.68 (0.50-0.92)	<0.01	1.05 (0.80-1.37)	0.70
**Perception: HP is a role model for patients and public**								
No	1		1		1		1	
Yes	1.13 (0.85-1.49)	0.37	1.06 (0.80-1.40)	0.65	1.95 (1.41-2.70)	<0.01	1.72 (1.17-2.52)	<0.01
**Perception: HP has a role in tobacco control**								
No	1		1		1		1	
Yes	1.39 (1.07-1.78)	0.01	1.21 (0.88-1.67)	0.23	1.59 (1.09-2.30)	0.01	1.08 (0.72-1.62)	0.72
**Received training about dangers of tobacco use**								
No	1		1		1		1	
Yes	1.56 (1.02-2.38)	0.03	1.62 (1.05-2.50)	0.02	1.54 (1.10-2.14)	0.01	1.44 (1.04-1.99)	0.02

OR = unadjusted odds ratios, CI = confidence intervals.

AOR = odds ratios adjusted for all independent variables.

### Support for anti-tobacco policies

Univariate analysis indicates that students were less likely to support anti-tobacco policies if they were current smokers (OR = 0.11, 95% CI (0.06-0.17), p-value <0.01); or smokers who attempted to quit smoking (OR = 0.27, 95% CI (0.18-0.39), p-value <0.01). However, they were more likely to support anti-tobacco policies if they perceived HP as a role model for patients and public (OR = 1.95, 95% CI (1.41-2.70), p-value < 0.01), perceived that HP has a role in tobacco control (OR = 1.59, 95% CI (1.09-2.30), p-value 0.01. After adjusting for all study variables, multivariate analysis indicates that students are less likely to support anti-tobacco policies if: they are smokers (OR = 0.21, 95% CI (0.08-0.56), p-value 0.01), and cigarette experimenters (OR = 0.42, 95% CI (0.26-0.70), p-value <0.01). They were more likely to support anti-tobacco policies if they are: aged 19 to 24 (OR = 1.84, 95% CI (1.12-3.04), p-value 0.01), perceive HP as role model for patients and public (OR = 1.72, 95% CI (1.17-2.52), p-value <0.01), and received training about dangers of tobacco use (OR = 1.44, 95% CI (1.04-1.99), p-value 0.02) (Table 2).

## Discussion

The study provides information about HP students’ attitudes towards tobacco control efforts. We found that almost ten percent of all HP students are smokers, and almost a quarter of them have experimented with cigarette smoking. Almost half of HP students were not aware of anti-tobacco policy, and one in ten of them did not support anti-tobacco policies. They were more likely to do so if they were current smokers or cigarette experimenters.

Although smoking prevalence among HPs appears to be on a decline among developed nations, the scenario is quite contrary among developing countries; where consistent evidence reports high smoking prevalence among HP students, and our results are comparable with these findings [16-18]. Research literature also shows that support towards anti-smoke policy at institutes and clinics depend greatly on the smoking status of health professionals. Students who were smokers showed weak support towards ban on smoking at institutes and public places. This complements with other studies in Malta and Greece that also suggests that health professional’s attitude towards anti-tobacco policies may be linked with their established smoking status [19,20].

Such findings have led to an international advocacy for curbing tobacco use among these future care givers, and undermine the need to focus this subset of population for future tobacco control efforts [12]. It was noted that the cigarette smoking HP students were at different stages of tobacco use: some were experimenters (ever used cigarettes), while others were current smokers, quitters or, smokers who attempted to quit smoking. The behavioral dynamics may be very different among individuals at different stages of smoking. We found that smokers showed least support for national anti-tobacco policies, followed by experimenters, and smokers who attempted to quit smoking. Unger et al. suggest that support for anti-tobacco policies declines as individuals move from never smoking to a smoking state; and inquire whether interventions aiming to increase support for anti-tobacco policies should discourage non- smoking individuals to initiate smoking, or encourage smokers to quit [9]. Perhaps we need more qualitative evidence in this regard in order to inform policy makers for improving support from HPs.

The finding of lack of awareness amongst them highlights an important behavioral aspect. If they lack awareness and support of anti-tobacco policies they might not advocate for the tobacco control efforts. Literature review shows that increased public support for anti-tobacco policies helps in enactment of anti-tobacco laws, and reducing smoking prevalence in the society [21]. The impact of such efforts may be greater if such support comes from the care givers in the society [22]. Moreover, smoking cessation is one of the most important, cost-effective preventive advices that health professionals can give to their patients, and has been regarded as the “gold standard” of preventive interventions [23]. Developing countries like ours may benefit from using such cost effective strategies, provided, the health professionals are prepared to take up the desired role in tobacco use cessation.

The results suggest that policy awareness was low among the HPs who were exposed to second hand smoke at work place or home. Having smoking exposure may contribute towards the development of positive attitude towards smoking, as a social norm and may increase the tendency to smoke in future [24]. This suggests that attitude towards anti-smoking policy represent one of the consequences of socio behavioral aspect associated with the high risk of smoking among HPs having a previous second hand smoke exposure.

The lack of awareness, and support regarding anti-tobacco policy was also associated with poor knowledge regarding tobacco hazards. HPs who had poor prior knowledge regarding tobacco hazards were also less likely to be aware of anti-tobacco policy at the work place, or support anti-tobacco bans. Our findings also emphasize the dire need for incorporating effective tobacco related knowledge in their graduate level trainings [25]. Additionally, with respect to support for these policies, it is of concern that students who were skeptic about themselves being portrayed as role models for the society, tend to be unsupportive of the anti-tobacco bans. Thus it is an important consideration that these trainings should also aim to color the perceptions of these students, which may contribute further towards successful patient counseling encounters in future, among them. Development and incorporation of tobacco control advocacy curricula may help increase awareness, and improve HP’s advocacy for anti-tobacco efforts [26,27].

This study reports association of various factors influencing anti-tobacco policy support and awareness among HP students in Pakistan. Our research study design has several limitations. First, the data is self-reported and creates a possibility of bias in the study but, all surveys were confidential and consistent research evidence suggests that it is a reliable method for data collection [28]. Secondly, the GHPSS data is collected from students enrolled in third year of graduate programs so there is a possibility that they might gain more knowledge regarding tobacco hazards in later years of training, and during their clinical practice. However, as compared to practicing HPs or final year HP students, it may be helpful to focus on third year HP students; as the information collected may later be used to improve the role of these students along their training period. Further, due to secondary analysis, we could not deal with the issue of non-responders. Lastly, this is a cross sectional study and direction of causality cannot be determined. There is a strong possibility that having a smoking status might influence attitude towards anti-tobacco policy; and more evidence from longitudinal research studies, may help to determine and deeply explore the causal associations. However, our study does indicate that attitude towards anti-tobacco policy is influenced by psychosocial variables and vary according to the smoking status of individuals.

Despite the above limitations this study provides useful information regarding the attitude of HP students towards awareness and support of anti-tobacco policies. We suggest that tobacco control program and workshops shall be integrated in health professions’ educational curricula and efforts shall be made to enhance the knowledge of HPs towards tobacco hazards. The HPs can play an important role in promoting awareness and implementation of anti-tobacco policies by effective counseling of their patients. Changing attitude of these future care givers is imperative for successful implementation of these policies, and thus should be considered as an important aspect during policy making.

## Conclusion

This study reports findings regarding HP students’ existing attitudes related to anti-tobacco policies. We suggest that various attitudinal aspects highlighted in this study may be taken into account for gaining full support of the future care givers with regard to tobacco control.

## Additional files

Additional file 1: Table S1. Coding plan for the selected study variables. Additional file 2: Table S2. Health professional students’ views about anti-tobacco policy and support by their current smoking status (n = 3612).
