# Supplementary material for: Awareness and support for anti-tobacco policies among health professional students in Pakistan: findings from the Global Health Professional Students Survey, 2011
Source: Subst Abuse Treat Prev Policy. 2015 Mar 8;10:7. doi: 10.1186/s13011-015-0001-x (PMC4357184; doi:10.1186/s13011-015-0001-x)
Supplement: Additional file 1: Table S1. — Coding plan for the selected study variables. [file 13011_2015_1_MOESM1_ESM.doc]

# Additional file 1: Table S1. Coding plan for the selected study variables

| **Variable; ID** | **Question In Codebook** |  | **Coding for analysis** |
| --- | --- | --- | --- |
| **Outcome Variables** |  |  |  |
| **Anti-tobacco policy awareness**  CR15 | Does your school have an official policy banning smoking (cigarettes, shisha, (Hukka), or other tobacco products) in school buildings and clinics? Options included: 1 = yes,for school building only, 2 = yes, for clinics only, 3 = yes, for both school buildings and clinics, 4 = no official policy |  | 0 = no (option 4) 1 = yes (option 1 to 3) |
|  |  |  |  |
|  |  |  |  |
|  |  |  |  |
|  |  |  |  |
| **Anti-tobacco policy support**  CR17, CR18, CR19, PKR21, PKR22, CR22 | Should tobacco (cigarettes, shisha (Hukka), or other tobacco products) sales to adolescents (persons younger than 18 years old) be banned? Options included: 1 = yes, 2 = no |  | Students who answered Yes to at least four questions were recoded as having support for anti-tobacco policies (1 = Yes) and all others were recoded as having no support (0 = No). |
|  |  |  |  |
|  |  |  |  |
|  | Should there be a complete ban of the advertising of tobacco products (cigarettes, shisha (Hukka), or other tobacco products)? Options included: 1 = yes, 2 = no |  |  |
|  |  |  |  |
|  |  |  |  |
|  | Should smoking any tobacco product (cigarettes, shisha (Hukka), or other tobacco products) be banned in restaurants? Options included: 1 = yes, 2 = no |  |  |
|  |  |  |  |
|  |  |  |  |
|  | Should smoking any tobacco product (cigarettes, shisha (Hukka), or other tobacco products) be banned in entertainment places/internet cafes? Options included: 1 = yes, 2 = no |  |  |
|  |  |  |  |
|  |  |  |  |
|  | Should smoking any tobacco product (cigarettes, shisha (Hukka), or other tobacco products) be banned in cafes? Options included: 1 = yes, 2 = no |  |  |
|  |  |  |  |
|  |  |  |  |
|  | Should smoking any tobacco product (cigarettes, shisha (Hukka), or other tobacco products) be banned in all enclosed public places? Options included: 1 = yes, 2 = no |  |  |
|  |  |  |  |
|  |  |  |  |
| **Independent Variables** | |  |  |
| **Smoking status**  CR1, CR3, CR29, CR30 | Have you ever tried or experimented with cigarette smoking, even one or two puffs? Options included: 1 = yes, 2 = no |  | Never smoker: HPs who had never tried cigarettes and did not smoke any cigarettes in last 30 days were recoded as 0; Ex-smoker: HPs who responded that they do not smoke now were recoded as 1; Experimenter: HPs who have not smoked any cigarette in last 30 days and had ever experimented with cigarettes were recoded as 2; Current smoker: HPs who had smoked for at least 1day during past 30 days were recoded as 3; Smokers who attempted to quit smoking: HPs who had ever tried to stop smoking cigarettes were recoded as 4. |
|  |  |  |  |
|  | During the past 30 days (one month), on how many days did you smoke cigarettes? Options included: 1 = 0 days, 2 = 1 or 2 days, 3 = 3 to 5 days, 4 = 6 to 9 days, 5 = 10 to 19 days, 6 = 20 to 29 days, 7 = all 30 days |  |  |
|  |  |  |  |
|  |  |  |  |
|  |  |  |  |
|  | Do you want to stop smoking cigarettes now? Options included: 1 = I have never smoked cigarettes, 2 = I do not smoke now, 3 = yes, 4 = no |  |  |
|  |  |  |  |
|  |  |  |  |
|  | During the past year, have you ever tried to stop smoking cigarettes? Options included: 1 = I have never smoked cigarettes, 2 = I did not smoke during the past year, 3 = yes, 4 = no |  |  |
|  |  |  |  |
|  |  |  |  |
|  |  |  |  |
| **Age in years (categorical)**  CR44 | How old are you? Options included: 1 = 14 years or younger, 2 = 15 to 18 years, 3 = 19 to 24 years, 4 = 25 to 29 years, 5 = 30 years or older |  | 0 = 15 to 18 |
|  |  |  | 1 = >18 up to 24 |
|  |  |  | 2 = > 24 (Those who reported to be <15 years old were excluded). |
|  |  |  |  |
| **Sex**  CR45 | What is your gender? Options included: 1 = female, 2 = male |  | 0 = female, 1 = male |
|  |  |  |  |
| **SHS exposure**  CR13, CR14 | During the past 7 days, on how many days have people smoked (cigarettes, shisha (Hukka), or other tobacco products) where you live, in your presence? Options included: 1 = 0 days, 2 = 1 to 2 days, 3 = 3 to 4 days, 4 = 5 to 6 days, 5 = all 7 days |  | 0 = no (for both questions) 1 = yes ( at least 1 yes for all options 2 to 5) |
|  |  |  |  |
|  |  |  |  |
|  |  |  |  |
|  | During the past 7 days, on how many days have people smoked (cigarettes, shisha (Hukka), or other tobacco products) in your presence, in places other than where you live? Options included: 1 = 0 days, 2 = 1 to 2 days, 3 = 3 to 4 days, 4 = 5 to 6 days, 5 = all 7 days |  |  |
|  |  |  |  |
|  |  |  |  |
|  |  |  |  |
| **Perception of HP as role model for patients and public**  CR24 | Do health professionals serve as "role models" for their patients and the public? Options included: 1 = yes, 2 = no |  | 0 = no, 1 = yes |
|  |  |  |  |
|  |  |  |  |
|  |  |  |  |
| **Perceived role of HP in tobacco control**  CR25 | Do health professionals have a role in giving advice or information about tobacco use cessation to patients? Options included: 1 = yes, 2 = no |  | 0 = no, 1 = yes |
|  |  |  |  |
|  |  |  |  |
|  |  |  |  |
| **Received training about dangers of tobacco use**  CR40 | During your (medical, dental, nursing, or pharmacy) school training, have you ever received any formal training in tobacco use (cigarettes, shisha (Hukka), or other tobacco products) cessation approaches to use with patients? Options included: 1 = yes, 2 = no |  | 0 = no, 1 = yes |
|  |  |  |  |
|  |  |  |  |
|  |  |  |  |
