# Supplementary material for: Awareness and support for anti-tobacco policies among health professional students in Pakistan: findings from the Global Health Professional Students Survey, 2011
Source: Subst Abuse Treat Prev Policy. 2015 Mar 8;10:7. doi: 10.1186/s13011-015-0001-x (PMC4357184; doi:10.1186/s13011-015-0001-x)
Supplement: Additional file 2: Table S2. — Health professional students’ views about anti-tobacco policy and support by their current smoking status (n = 3612). [file 13011_2015_1_MOESM2_ESM.doc]

**Additional file 2: Table S2. Health professional students’ views about anti-tobacco policy and support by their current smoking status (n = 3612)**

|  |  |  |  | |  | Never -smoker | Ex-smoker | Experimenter | Current Smoker | Smokers who –attempted to quit |
| --- | --- | --- | --- | --- | --- | --- | --- | --- | --- | --- |
|  |  |  |  | |  | n = 2820 | n = 396 | n = 965 | n = 391 | n = 349 |
|  |  |  |  | |  | n (%) | n (%) | n (%) | n (%) | n (%) |
| **Anti-tobacco policy awareness** | |  | |  |  |  |  |  |  |  |
| Does your school have an official policy banning smoking (cigarettes, shisha, hukka, or other tobacco products) in school buildings and clinics? | | | | | Yes | 1486 (52.7) | 223 (56.3) | 570 (59.1) | 255 (65.2) | 207 (59.3) |
|  |  |  |  |  | No | 1334 (47.3) | 173 (43.7) | 395 (40.9) | 136 (34.8) | 142 (40.7) |
|  |  |  |  |  |  |  |  |  |  |  |
|  |  |  |  | |  |  |  |  |  |  |
| **Anti-tobacco policy support** | |  |  | |  |  |  |  |  |  |
| Should tobacco (cigarettes, shisha, hukka, or other tobacco products) sales to adolescents younger than 18 years old) be banned? | | | | | Yes | 2562 (90.9) | 361 (91.2) | 814 (84.4) | 313 (80.1) | 297 (85.1) |
|  |  |  |  |  | No | 258 (9.1) | 52 (8.8) | 151 (15.6) | 78 (19.9) | 52 (14.9) |
|  |  |  |  |  |  |  |  |  |  |  |
| Should there be a complete ban of the advertising of tobacco products (cigarettes, shisha, hukka, or other tobacco products)? | | | | | Yes | 2626 (93.1) | 353 (89.1) | 787 (81.6) | 289 (73.9) | 287 (82.2) |
|  |  |  |  |  | No | 194 (6.9) | 43 (10.9) | 178 (18.4) | 102 (26.1) | 62 (17.8) |
|  |  |  |  |  |  |  |  |  |  |  |
| Should smoking any tobacco product (cigarettes, shisha, hukka, or other tobacco products) be banned in restaurants? | | | | | Yes | 2661 (94.4) | 346 (87.4) | 772 (80.0) | 262 (67.0) | 273 (78.2) |
|  |  |  |  |  | No | 159 (5.6) | 50 (12.6) | 193 (20.0) | 129 (33.0) | 76 (21.8) |
|  |  |  |  |  |  |  |  |  |  |  |
| Should smoking any tobacco product (cigarettes, shisha, hukka, or other tobacco products) be banned in entertainment places/internet cafes? | | | | | Yes | 2626 (93.1) | 319 (80.6) | 712 (73.8) | 235 (60.1) | 242 (69.3) |
|  |  |  |  |  | No | 194 (6.9) | 77 (19.4) | 253 (26.2) | 156 (39.9) | 107 (30.7) |
|  |  |  |  |  |  |  |  |  |  |  |
| Should smoking any tobacco product (cigarettes, shisha, hukka, or other tobacco products) be banned in cafes? | | | | | Yes | 2635 (93.4) | 308 (77.8) | 709 (73.5) | 242 (61.9) | 245 (70.2) |
|  |  |  |  |  | No | 185 (6.6) | 88 (22.2) | 256 (26.5) | 149 (38.1) | 104 (29.8) |
|  |  |  |  |  |  |  |  |  |  |  |
| Should smoking any tobacco product (cigarettes, shisha, hukka, or other tobacco products) be banned in all enclosed public places? | | | | | Yes | 2692 (95.5) | 358 (90.4) | 831 (86.1) | 307 (78.5) | 302 (86.5) |
|  |  |  |  |  | No | 128 (4.5) | 38 (9.6) | 134 (13.9) | 84 (21.5) | 47 (13.5) |
|  |  |  |  |  |  |  |  |  |  |  |
